# Histological ovarian features and hormonal determinations in assigned females at birth transgender individuals according to different testosterone preparations

**DOI:** 10.3389/fendo.2024.1458846

**Published:** 2024-10-28

**Authors:** Aina Borrás, Yasmina Barral, Francesc Fabregues, Gemma Casals, Mireia Mora, Aida Orois, Marta Méndez, Adela Saco, Anna Goday, Dolors Manau

**Affiliations:** ^1^ Clinical Institute of Gynecology, Obstetrics, and Neonatology, Hospital Clinic, Barcelona, Spain; ^2^ Institut d’Investigacions Biomèdiques August Pi i Sunyer (IDIBAPS), Gynecology, Human Reproduction and Women’s Health Group, Barcelona, Spain; ^3^ Endocrinology and Nutrition Department, Hospital Clínic, Universitat de Barcelona, Centro de Investigación Biomédica en Red. Diabetes y Enfermedades Metabólicas Asociadas (CIBERDEM), Barcelona, Spain; ^4^ Deparment of Pathology, Hospital Clinic, University of Barcelona, Barcelona, Spain

**Keywords:** hormone treatment, histological ovarian features, different testosterone preparations, transgender, hormonal profile

## Abstract

**Introduction:**

Distinct androgen formulations have been used as gender-affirming hormone treatment, but little is known about the specific changes that may occur in the ovary depending on the testosterone preparation used. The study aims to evaluate the histological modifications of the ovarian tissue and the hormonal changes after gender-affirming surgery based on the testosterone preparation employed, such as testosterone cypionate or undecanoate.

**Design:**

Unicenter transversal cohort study.

**Materials and methods:**

Sixty transmasculine persons before and after gender-affirming surgery. A histological examination of the ovaries was conducted, including the follicular population and the characterization of the ovarian stroma. Hormonal status (testosterone, estradiol, FSH, and LH) were also assessed before and after the procedure.

**Results:**

The median age of participants was similar between the two groups (27.9 vs. 26.7 years, p = 0.27). There were no differences in all hormonal determinations before gender-affirming surgery between the groups. After surgery, FSH levels increased significantly, especially in the testosterone undecanoate group compared to the cypionate group (72.3 vs. 38.3 U/L, p = 0.02), consistent with LH determinations (43.0 vs. 23.4 U/L, p = 0.02). However, no regimen modification was required for any individual. No statistical differences were observed in any parameter concerning the follicular population, nor were there any variances in the thickness of the tunica albuginea (p = 0.85) or the proportion of luteinized stromal cells. Nevertheless, there was a tendency toward increased luteinization in the testosterone cypionate group (88.2% vs. 76.9%, p > 0.05).

**Conclusions:**

In a cohort of transmasculine individuals using different androgen preparations, histological analysis of ovarian tissue revealed comparable findings. Both groups exhibited similar follicular populations and comparable modifications in stromal tissue. However, significant differences were observed in hormonal profiles, although no modification in testosterone dosage was needed.

## Introduction

Hormone treatment is essential for individuals experiencing gender incongruence, as it helps to develop secondary sexual characteristics according to their affirmed gender. Transgender and gender-diverse (TGD) people assigned female at birth initiate gender-affirming hormone treatment (GAHT) with testosterone to achieve this goal and maintain sex hormone levels within the normal male range (1). In this study, the term transmasculine people is used to describe TGD people assigned female at birth but who identify otherwise.

Some clinical investigations have confirmed the efficacy of several distinct androgen formulations in stimulating masculinization among transmasculine individuals (2). These regimens adhere to the fundamental principle of hormone replacement therapy for cisgender male hypogonadism, and different androgen formulations with specific pharmacokinetic profiles have been documented for achieving long-term transition objectives among transmasculine individuals. Currently, available applications include transdermal as well as intramuscular testosterone esters (1, 2). In recent studies, subcutaneous testosterone injections have been introduced as an alternative effective method, but its use is still limited in our environment (3, 4).

Nowadays, intramuscular testosterone esters are the most widely used androgen preparations due to their tolerance, availability, and the interval of injections necessary to achieve testosterone levels in the cisgender male reference range. Long-acting testosterone formulations such as enanthate or cypionate (100 – 250 mg intramuscular every 2 – 3 weeks) or testosterone undecanoate (1000 mg intramuscular every 10 - 16 weeks) allow to achieve these goals. It has also been reported that higher testosterone doses of enanthate or cypionate (250 mg every 2 weeks) are associated with earlier physical changes compared with lower doses (125 mg every 2 weeks or 250 mg every 3 weeks) but, still, outcomes are no longer significantly different after 6 months (5).

The initiation of GAHT with the different testosterone preparations relies on the drug financing within the Public Health System of our country, and both options are viable for initiating and facilitating an individual’s transition to their self-perceived gender. Furthermore, some transmasculine people, after starting hormonal therapy, will consider hysterectomy and bilateral oophorectomy to achieve a complete transition and alleviate the dysphoria they experience, or as a prerequisite for further genital surgeries like phalloplasty or metoidioplasty. Moreover, considerations for such surgeries may also arise due to symptom-related problems such as pelvic pain related to endometriosis or abnormal uterine bleeding (6, 7).

There is limited understanding of the specific and differential changes that may occur depending on the testosterone preparation used, such as testosterone cypionate or undecanoate. Therefore, the study aims to evaluate the histological modifications of the ovarian tissue after gender-affirming surgery (GAS) based on the testosterone preparation employed. Secondary objectives involve characterizing the hormonal changes following surgery in transmasculine individuals, considering the long-term hormonal treatment preceding GAS. We hypothesize that histological changes will be comparable across both groups after two years of treatment with different testosterone preparations. However, discrepancies may exist in hormonal parameters based on the type of testosterone administered.

## Materials and methods

### Study design

This study was conducted as a Unicenter transversal cohort investigation approved by the review board of Hospital Clínic de Barcelona (registration number HCB/2021/0250). A cohort of 60 transmasculine individuals undergoing testosterone therapy for over 24 months participated between January 2016 and January 2021 before gender-affirming surgery, which included a total laparoscopic hysterectomy and bilateral oophorectomy. No significant side effects were reported during hormone therapy in any of the individuals. GAS was performed at the individual’s request to reduce the dysphoria they experienced.

Transmasculine persons were enrolled in the study when GAS was scheduled. Histological examination of the ovaries was conducted post-surgery, along with an assessment of hormonal status before and after the procedure. None of the transmasculine individuals were using any contraceptives at baseline or during the study, nor GnRHA agonists. Before hormonal therapy, all had regular menstrual cycles according to their medical history, except for four patients, who presented with oligomenorrhoea, hirsutism, and hormone determinations compatible with PCOS. Tobacco consumption or the presence of other medical comorbidities such as hypothyroidism or chronic pelvic pain were assessed in both cohorts. The transmasculine people received intramuscular testosterone therapy with testosterone undecanoate (Reandron^®^ 1000 mg, Grunenthal Pharma, Spain) every 10 - 16 weeks in 26 cases, or testosterone cypionate (Testex prolongatum^®^ 250 mg/2 ml, Desma Laboratory, Spain) every 14 - 28 days in 34 cases, for a minimum duration of 24 months. Changes in secondary sexual characteristics were observed approximately after 2 months of treatment and, in all cases, amenorrhea was achieved after 3-6 months of GAHT. The availability of funding for the drug within the public health system during the study recruitment period primarily determined the choice of testosterone formulation. Aligned with international guidelines, such as the World Professional Association for Transgender Health recommendations (WPATH), laboratory tests were performed every three months after a new testosterone regimen was initiated for a year. Subsequently, these tests transition to biannual intervals once the maintenance dose is achieved. Testosterone cypionate monitoring was performed between the dosing interval, while monitoring treatment with testosterone undecanoate measurement was performed just before the next injection, approximately 1 week before.

### Study parameters

For all participants, physical and biochemical studies included weight, height, and body mass index measurements. Additionally, a blood sample was taken to determine serum hormone levels (at any time of the ovarian cycle, not specifically in the early follicular phase) and, subsequently, a second blood sample was obtained between 3 - 4 months following the surgical procedure.

### Hormonal analysis

Biochemical parameters were assessed in serum using standard methodologies at the Core Laboratory of our center. Results were reported based on cisgender male reference ranges. Total testosterone levels were determined via chemiluminometric immunoassay (Testosterone II, Elecsys) by Roche, Mannheim, Germany, with a lower limit of quantification (LOQ) of 12 ng/dl and an interassay coefficient of variation of < 5%. The normal range for total testosterone was 275.0 – 850.0 ng/dl. Estradiol, follicle-stimulating hormone (FSH), and luteinizing hormone (LH) levels were assessed using chemiluminometric immunoassays performed on the ATELLICA Immunochemistry analyzer (Siemens Healthineers, Tarrytown, NY, USA). The interassay coefficient of variation was < 5% for estradiol (with a LOQ of 18.8 pg/ml), < 10% for FSH (with a LOQ of 0.55 mIU/ml), and < 5% for LH (with a LOQ of 1.44 mIU/ml).

### Ovarian histological study

Regarding the ovarian histopathological study, specimens from the hysterectomy and oophorectomy were transported to the laboratory and subjected to macroscopic examination. Surgical pieces were weighed, and measured, and ovarian volumes were calculated using the ellipsoid formula (length × width × height × 0.523). Subsequently, the ovaries were immediately processed by a gynecological pathologist. A section encompassing the ovary’s largest diameter was excised and fixed in 10% formalin for histological analysis. This procedure was performed for both ovaries of each individual. The sections were then embedded in paraffin, and 3-μm-thick sections were prepared. These sections were stained with hematoxylin and eosin using standard techniques and were examined by a specialized gynecological pathologist.

The ovarian follicular developmental stages were assessed following the criteria outlined by Gougeon and Chainy (8). Primordial follicles were identified as those measuring 30 – 60 μm in diameter and featuring a single layer of flat granulosa cells surrounding the oocyte. Primary follicles were characterized by a layer of cuboidal cells and a diameter exceeding 60 μm. Secondary follicles were described as having more than two layers of cuboidal cells with a diameter ranging from 0.12 to 0.2 mm. In contrast, early antral follicles were distinguished by a follicular antrum measuring 0.12 to 2mm in diameter. Antral follicles wider than 2 mm in diameter and atretic follicles were also evaluated. Follicles were individually counted within each category and were categorized into sections containing the nucleus to prevent duplication in counting.

**Figure 1:**
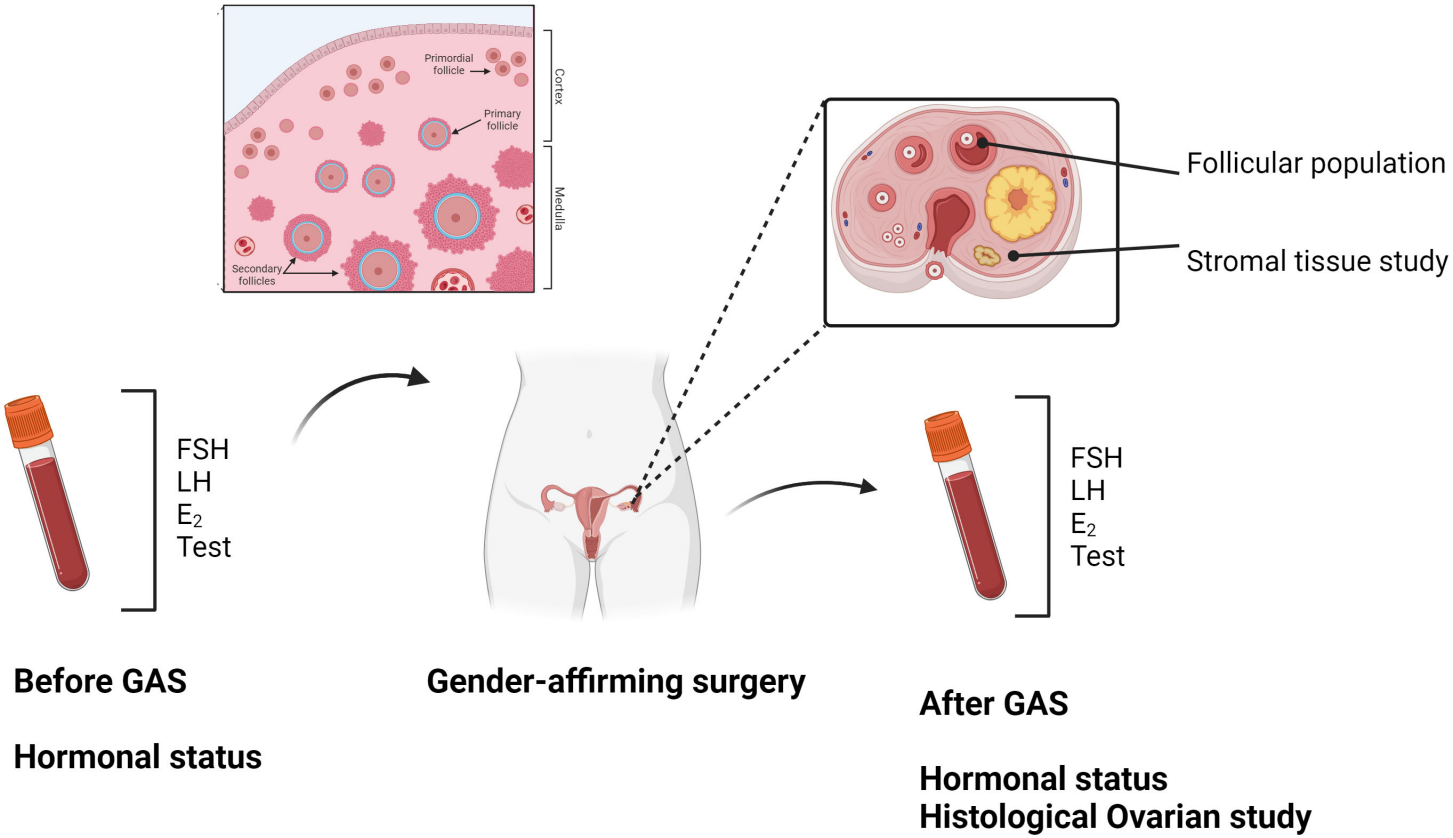
Created in BioRender. Borras Capo, A. (2024) BioRender.com/d20i488.

Changes in the ovarian stroma were evaluated, focusing primarily on the collagenization and thickening of the tunica albuginea, stromal hyperplasia, and the luteinization of the stromal cells (9). The thickness of the ovarian cortex along the entire longitudinal section of the ovary was examined and measured, determining the thickest and thinnest points of the cortex section in both ovaries. The presence of stromal hyperplasia, luteinization of stromal cells, and the number of atretic follicles in all ovarian tissue samples were also assessed. **Figure 1** provides an overview of the study's methodology.

### Statistical analysis

Statistical analyses were performed using IBM SPSS Statistics 23.0 (IBM Corp., New York, USA). Descriptive statistics were computed, including medians with ranges for continuous variables and percentages for categorical variables.

Normality was assessed using the Shapiro–Wilk test. Depending on the data type and distribution, univariate analysis was conducted using the Student’s T-test or the Mann–Whitney U test to calculate differences between continuous variables.

Histological characteristics including stromal hyperplasia, stromal luteinization, and thickness of the tunica albuginea were assessed for presence or absence, and the ratio of cases exhibiting these characteristics was calculated. The number and percentage of follicles at various stages of development were also determined. Chi-square analysis was conducted to compare these parameters, with the Fisher exact test utilized in case of any infrequent response. A p-value of < 0.05 was considered to denote a statistically significant difference.

## Results

The median age of participants was 27.9 years old (interquartile range [IQR] 23 - 32) in the testosterone undecanoate group, and 26.7 years old (interquartile range [IQR] 23 - 34) in the testosterone cypionate group, comparable (p = 0.27). Both groups were largely within a healthy BMI range with a median of 22.4 kg/m^2^ (IQR 19.8 - 24.8) in the testosterone undecanoate group and 23.1 kg/m^2^ (IQR 18.9 – 25.4) in the testosterone cypionate group (p = 0.42), and more than a quarter of the cohort were smokers. The median time on testosterone treatment was 27.9 months (IQR 25 – 32) in the testosterone undecanoate group and 27.2 months (IQR 24 – 32) in the testosterone cypionate group (p = 0.6), before GAS. The baseline characteristics of both groups are described in **Table 1**, along with the analytical parameters before and after GAS.

**Table 1:** Clinical and hormonal characteristics in transmasculine people (before and after gender-affirming surgery).

Parameter	Testosterone undecanoate (N=26)	Testosterone cypionate (N=34)	P value
	Median (IQR) or No. (%)	Median (IQR) or No. (%)	
**Age (years)**	27.91 (23-32)	26.72 (23-34)	0.27
**Smokers**	8 (30.7%)	9 (26.5%)	0.78
Medical comorbidities:			
**Hypothyroidism**	3 (11.5%)	5 (14.7%)	0.75
**Polycystic ovarian syndrome**	2 (7.7%)	2 (5.8%)	0.79
**Pelvic chronic pain**	5 (14.7%)	8 (23.5%)	0.74
**FSH presurgery (U/L)**	4.17 (0.96-6.75)	4.98 (3.8-6.3)	0.61
**LH presurgery (U/L)**	7.82 (4.5-10.5)	7.14 (42-8.9)	0.66
**E_2_ presurgery (pg/ml)**	59.52 (33.5-76.5)	74.38 (52.5-94.5)	0.27
**TT presurgery(ng/ml)**	728.32 (480.4-956.6)	792.91 (601.3-952.1)	0.45
**FSH postsurgery (U/L)**	72.29 (31.8-107.0)	38.31 (5.5-59.4)	0.02*
**LH postsurgery (U/L)**	43.03 (17.4-72.5)	23.42 (2.2-36.9)	0.02*
**E_2_ postsurgery (pg/ml)**	38.51 (31.2-48.7)	40.32 (31.2-52.5)	0.16
**TT postsurgery (ng/ml)**	615.61 (437.1-823.2)	582.14 (371.0-666.8)	0.65

Acronyms: BMI, body mass index; FSH, follicle-stimulating hormone; LH, luteinizing hormone; E_2_, estradiol; TT, total testosterone. *p<0.05, significant differences.

No differences were found in hormonal determinations between the two groups before gender-affirming surgery. Similar testosterone levels were observed before surgery: 728.3 ng/dl (IQR 480.4 - 956.6) in the testosterone undecanoate group versus 792.9 ng/dl (IQR 601.3 - 952.1) in the cypionate group (p = 0.45). After GAS, testosterone levels decreased in both groups, with no differences found between them. Similarly, lower estradiol levels were observed in both groups after GAS, with no differences globally [38.5 pg/ml in the testosterone undecanoate group (IQR 31.2-48.7) versus 40.3 pg/ml in the cypionate group (IQR 31.2-52.5)], p = 0.16. Significant differences were observed when comparing estradiol levels before and after surgery in the testosterone cypionate group, with a marked decrease in E2 levels following bilateral oophorectomy: 74.4 pg/ml (IQR 52.5-94.5) vs 40.3 pg/ml (IQR 31.2-52.5), p = 0.04. No statistical differences were found in pre- and post-surgery estradiol levels in the testosterone undecanoate group, but a notable decrease was also observed (p = 0.06).

**Figure 2:**
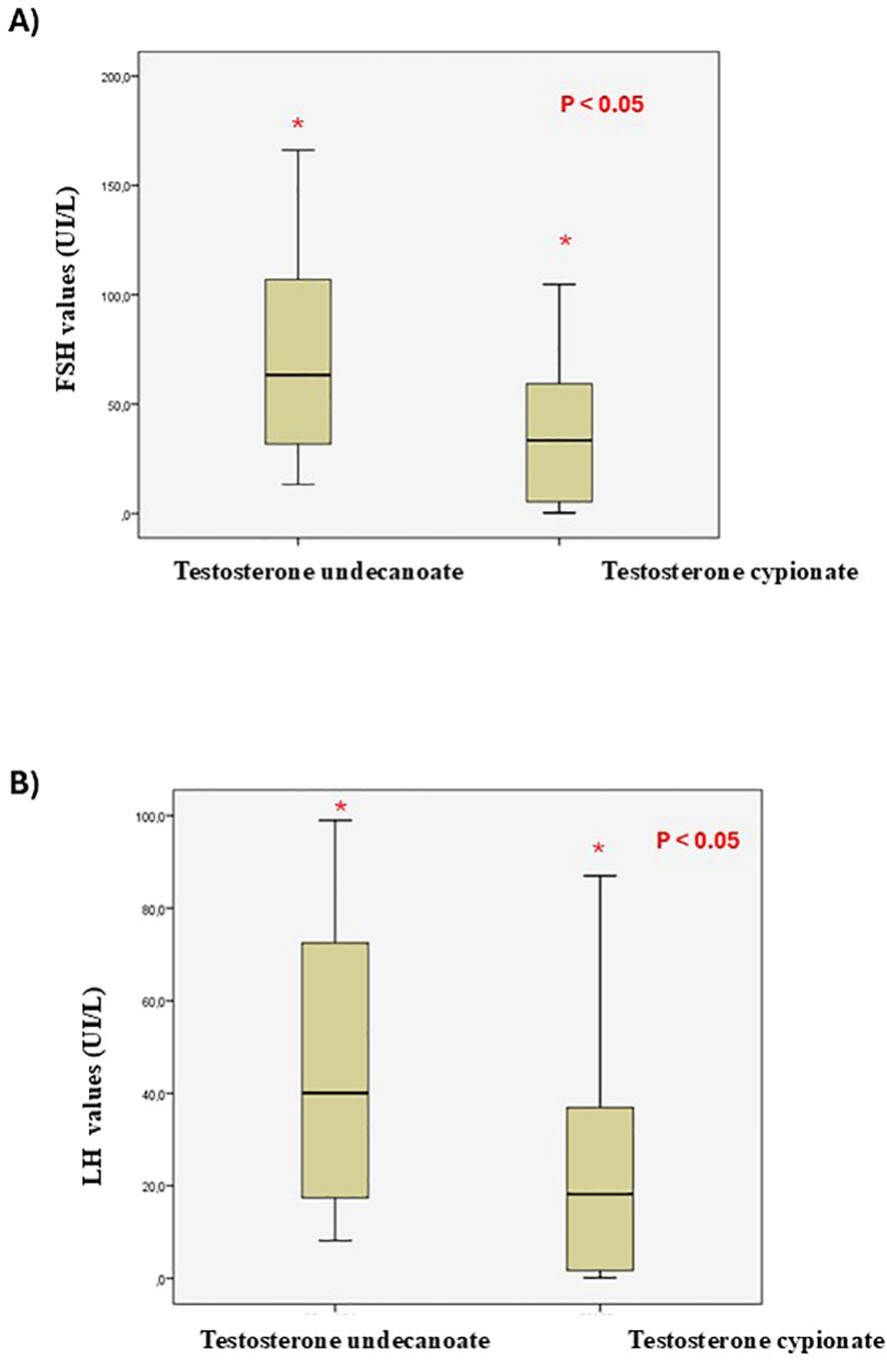
Mean change in laboratory values of FSH **(A)** and LH **(B)** levels (Box plot figures).

As expected, LH and FSH levels increased significantly after the surgery in both groups. A considerable heterogeneity has been noted in the values derived from hormonal assessments, revealing notable distinctions between the two groups: elevated FSH levels were particularly prominent in the testosterone undecanoate group [72.3 U/L (IQR 31.8 - 107.0)] compared to the cypionate group [38.3 U/L (IQR 5.5 - 59.4)] (p = 0.02), consistent with LH determinations: 43.0 U/L (IQR 17.4 - 72.5) versus 23.4 U/L (IQR 2.2-36.9) (p=0.02) (**Figures 2A, B**). Nevertheless, no regimen modification was required in either of the two treatment groups.

**Figure 3:**
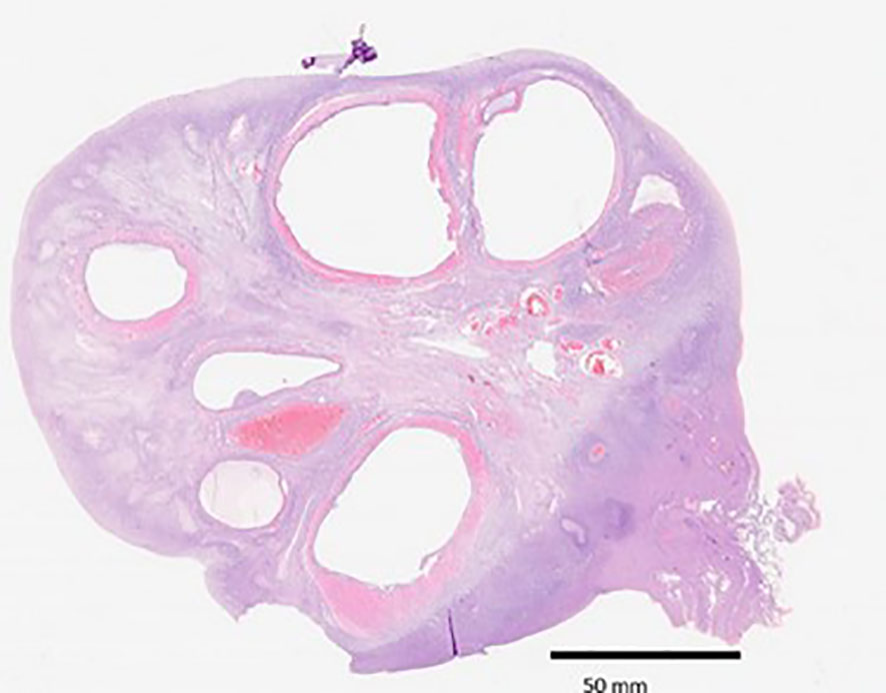
An ovarian hemisection showing follicles in different states of maturation (HE, 10x).

**Figure 4:**
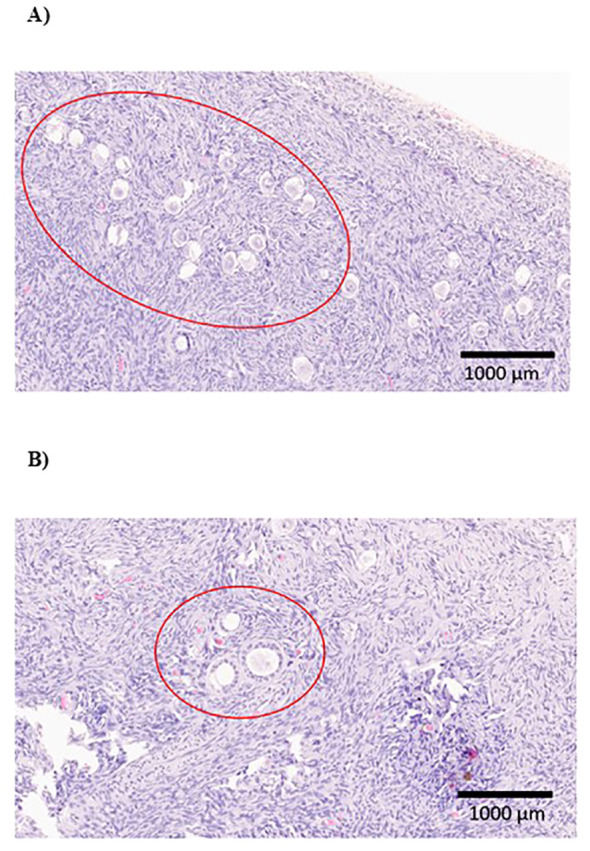
**(A)** Cluster of primordial follicles in an ovarian tissue sample from a transmasculine person treated with testosterone undecanoate (200x). **(B)** Histological slide of ovarian tissue from a transmasculine individual treated with testosterone cypionate, showing primary and secondary fol·licles (200x).

The histological characteristics of the samples are presented in **Table 2**. Primordial follicles were detected in all samples, showing a wide variability in terms of quantity (**Figure 3**). However, follicles in more advanced developmental stages were not universally observed (over 80% in both groups, p > 0.05). Additionally, no statistical differences were noted in any parameters concerning the follicular population based on the administered treatment (**Figures 4A, B**). Regarding the ovarian stroma, no variances were identified in the thickness of the tunica albugínia (p = 0.85) or the proportion of luteinized stromal cells. Nevertheless, a clear tendency toward increased luteinization of stromal cells was noted in the testosterone cypionate group (88.2% versus 76.9%, p > 0.05).

**Table 2:** Histological ovarian evaluation according to the testosterone preparation in transmasculine people.

Histological Features		TT undecanoate (N=26)	TT cypionate (N=34)	P values
FOLLICLE STAGE		Median (IQR) or No (%)	Median (IQR) or N. (%)	
**Primordial**		17.31 (4.25-28)	36.62 (6-67)	0.08
**Primary**		1.38 (0-2)	1.88 (0-2.2)	0.54
**Secondary**		0.13 (0-1)	0.66 (0-1)	0.13
Antral follicles				
**Small Antral**		1.19 (0-2)	1.23 (0-2)	0.96
**Antral**		1.71 (0-2.75)	2.0 (0-3.25)	0.53
**Atresic**		2 (0-3)	1.68 (0-3)	0.6
**Thickness tunica albugínia**		0.55 (0.4-0.68)	0.54 (0.4-0.69)	0.85
**Stromal hyperplasia**		2 (7.7%)	3 (8.8%)	0.88
**Luteinization stromal cells**		22 (76.9%)	30 (88.2%)	0.91

## Discussion

This study provides a comprehensive comparison of ovarian changes observed in tissue following gender affirmation surgery (GAS), depending on the testosterone formulation administered, along with hormonal changes assessed before and after surgery. It is the first study to evaluate these parameters globally and compare the follicular and stromal ovarian structure.

According to data from the US Transgender Health Survey, an informal survey of approximately 30.000 transmasculine adults, over 70% of them indicated they had undergone hormone therapy at some point (10). As an important component of the needs of this group, clinicians may prescribe testosterone to achieve the desired secondary sexual characteristics of the self-perceived gender, tailoring the hormone regimens, based on the World Professional Association for Transgender Health (WPATH) recommendations, along with other professional organizations (11). Various sex hormone formulations, delivery methods, and dosages exist, allowing for customization based on patient choice, financial considerations, and the safety profiles of specific medications (1, 12). On the other hand, gynecological gender-affirming surgery can aid in the transition process by removing internal reproductive organs, thereby facilitating alignment with one’s gender identity (7).

It is worth noting that there is a lack of data guiding clinicians in the provision of testosterone therapy specifically for transmasculine persons (13, 14). The majority of GAHT protocols are extrapolated from the experience with androgen replacement in hypogonadal cisgender men (15), and several studies have indicated similarities in hormone profiles between transmasculine people and hypogonadal cisgender men receiving exogenous testosterone (16, 17). However, GAHT objectives diverge between these two populations, highlighting the necessity for further research into the most suitable protocols for transmasculine individuals. The predominant mode of administration involves intramuscular injections of testosterone esters, such as testosterone cypionate. However, alternative options such as intramuscular injections of longer-acting testosterone undecanoate are frequently employed (18, 19).

Testosterone cypionate is commercially dissolved in cottonseed oil, elicits peak levels approximately 72 hours post-injection, and gradually declines over the ensuing 10 to 14 days, exhibiting an exponential decrease and returning to baseline levels by day 21. Meanwhile, testosterone undecanoate is dissolved in refined castor oil, and, conversely, showcases an appealing pharmacokinetic profile with a half-life of 34 days and intervals between injections extending up to 13 weeks (4, 20–22). These depot formulations provide more stable plasma levels, whereas testosterone cypionate exhibits greater fluctuations, making it challenging to achieve the therapeutic target easily (4, 20–22). There are no randomized clinical trials that rank the different testosterone treatment types, so the choice of hormonal regimen in our center depends on the availability of financing within the healthcare system. During certain periods lasting several months, testosterone undecanoate was unavailable, resulting in distinct populations solely based on the type of testosterone administered. The individuals included in the study were similar in terms of their average age and the lack of chronic conditions, such as diabetes or immunological disorders.

Overall, no differences were found in the ovarian follicular population or stromal tissue based on the administered testosterone formulation, despite variances in the pharmacokinetic profiles of both testosterone esters. Nonetheless, post-surgical hormonal assessments showed significant differences between both groups, although no adjustment in the administered testosterone dose was required.

Various studies have examined the ovarian follicular population and stromal changes in transmasculine individuals. However, it’s important to note significant variability in the design of histological studies and the duration of testosterone administration (23–26). Furthermore, except for a recent publication by Asseler et al., no differences have been previously reported in ovarian studies based on the type of testosterone administered (27). This group evaluated the presence of antral and ovulatory follicles in a cohort of transmasculine populations undergoing different kinds of testosterone therapy post-surgery. They observed no differences in the presence or number of small antral follicles. However, other follicle populations, such as primordial follicles or stromal tissue, were not assessed, and the duration of gender-affirming hormone therapy (GAHT) was not specified in the study.

Ovarian follicular and stromal parameters have also been compared between fertile cisgender women and transmasculine persons in previous studies, revealing a similar cortical distribution to that observed in the current research (8, 28, 29). A comparison between these two populations (with a historical control group) was previously conducted by our group, demonstrating a maintained follicular population in the transmasculine cohort after two years of testosterone therapy, indicating no follicular loss (25). Additionally, the results of the present study confirm that the quantity and distribution of follicles remain constant regardless of the type of testosterone preparation used.

The differences in the pharmacokinetic profiles of both testosterone formulations may explain the differential effects observed in ovarian tissue in the study, particularly the changes in ovarian stroma. However, continuous therapy use without cessation for more than two years could have mitigated these changes, which might have been more pronounced during the initial months when testosterone level adjustments could have been greater. Additionally, these changes, although minimal, may have implications for processes such as fertility preservation, the timing of testosterone cessation for ovarian cycle recovery, or, in the context of ovarian tissue cryopreservation, the follicles’ potential for subsequent *in vitro* maturation process (24, 30, 31).

Regarding hormonal modifications according to different testosterone preparations, Pelusi et al. compared the effects of three different testosterone formulations - testosterone enanthate, gel, and undecanoate - in a 45-transmasculine population cohort -, at baseline and after 12 months of treatment. Interestingly, no differences were observed regarding short-term safety, adherence, metabolic indicators such as testosterone levels, FSH/LH/estradiol levels, or overall life satisfaction (32). On the other hand, Kumar et al. reported a statistically significant decrease in estradiol levels after bilateral oophorectomy (after one year of GAHT) compared to presurgical levels. They did not find a significant difference in total testosterone levels before and after oophorectomy, showing results similar to those reported in our cohort. They suggest that oophorectomy further attenuates estradiol levels below what is achieved by high-dose exogenous testosterone alone (33). However, in this last study, the type of testosterone administered was not specified, and the modification in FSH and LH levels in the studied population was not evaluated. Regarding this point, as seen in routine clinical practice, following gender-affirming surgery (GAS) and with the maintenance of a stable testosterone dose, an increase in gonadotropin levels (FSH and LH) is commonly observed. This rise is likely attributable to the absence of ovarian hormone secretion, particularly estrogens. Although menstrual cycles are absent, ovarian estrogens may still contribute to maintaining gonadotropin levels within the normal/low range (reference values for cisgender women). After GAS, the absence of these ovarian hormones may be sufficient to cause an elevation in gonadotropin levels, despite stable testosterone levels compared to pre-surgical values.

Additionally, these contrasts in the pharmacokinetics of testosterone preparations may account for the observed changes in hormonal assessments following surgery in both population groups. Before surgery, there were no differences in FSH, LH, or testosterone levels between the two groups. However, following GAS, FSH and LH levels differed significantly, with considerable variability observed among the transmasculine individuals studied, but did not entail differences in symptoms or the need to modify testosterone dose. Testosterone cypionate had a shorter half-life, which might lead to higher testosterone peak levels following administration, resulting in greater suppression of the pituitary axis, followed by a gradual decline. The timing of blood tests might yield more variable and lower gonadotropin values than testosterone undecanoate, a more stable, long-acting preparation that likely causes less suppression of the pituitary axis. Regarding this point, Grimstad et al, also described that those transmasculine individuals who underwent bilateral oophorectomy were no more likely to change their testosterone dose than those who did not undergo the GAS (34). Other authors also described this affirmation (35). This data is important because androgen therapy typically requires lifelong continuation to sustain the achieved masculinization and prevent symptoms associated with hypogonadism, such as vasomotor issues or osteoporosis, mainly when GAS is performed (36). Therefore, maintaining the dose to keep adequate testosterone levels appears to be independent of the hormonal levels observed after surgery, which vary widely depending on the testosterone preparation used.

The complete effect of testosterone on reproductive capacity remains unclear in the long term. At the moment, national and international medical organizations recommend counseling on fertility preservation options before initiating testosterone treatment, and above all, if the GAS is raised (2, 37, 38). This recommendation is made due to insufficient data to assure reproductive potential post-testosterone therapy, especially following bilateral oophorectomy, which results in permanent sterility. Conversely, there is increasing interest in the impact of various testosterone regimens on different health aspects in the transmasculine population, such as bone health. A recent review concluded that most hormonal preparations maintained or increased actual bone mineral density among transmasculine adults taking GAHT for at least 12 months from the outset. Despite the different bone turnover markers in the studies, a direct comparison of the influence of selected hormonal preparations on these clinical laboratory measures could not be made. They highlight the need for broader and standardized studies to evaluate these changes in transmasculine adults taking standardized GAHT regimens (12). Currently, no studies, except for the present one, have assessed the effects of different testosterone regimens on reproductive parameters, including changes in ovarian histology, the recovery of the menstrual cycle after discontinuation of GAHT, or pregnancy rates (both spontaneous and via ART) in transmasculine persons or their partners.

We recognize that our study has several limitations. The absence of a control group in the ovarian histological study and the small sample size are the greatest limitations of this study, warranting caution in generalizing the findings. However, previous studies based on the same line had similar sample sizes, and the observed hormonal modifications are consistent with ours (23, 25, 27, 34). Concerning the control group, the absence of an age-matched control group in the histological assessment is noteworthy. At our hospital, only a limited number of ovarian tissue samples can be procured from oncological cisgender female patients, and some samples are obtained from cisgender fertile women with benign or malignant ovarian masses. However, obtaining ovarian samples from non-oncological cisgender females with unilateral or bilateral oophorectomy for conditions like uterine fibroids or other benign gynecological diseases was not feasible (39). Additionally, we have presented data from the literature demonstrating results consistent with those observed in our study. Also, several studies have assessed life satisfaction with testosterone treatment, reporting high satisfaction levels across all subjects regardless of the formulation used. This indicates an improvement in the quality of life and mood among transmasculine persons following the initiation of GAHT (32, 40). However, our study did not investigate which aspects of life changes were more or less satisfactory for these individuals, depending on the different testosterone formulations used. Finally, assessing the anti-Müllerian hormone (AMH) and its variations across the testosterone groups would have provided valuable insights for the study. Few determinations have been performed in the sixty transmasculine people owing to difficulties in availability, and most of them just before surgery and in the same group. Little is known about its modifications when GAHT is initiated, and conflicting results have been reported (24, 25, 41). These aspects require further and in-depth research.

Despite its limitations, the present study thoroughly assesses ovarian histology, including both follicular and stromal components, in a significant group of transmasculine individuals. It takes into account the different testosterone preparations used in our environment and relates this follicular population to pre- and post-surgical hormonal levels. However, it is crucial to acknowledge that further studies, with larger sample sizes, are imperative to corroborate and refine these findings.

On the other hand, it is important to highlight that in our country fertility preservation before GAHT is currently an option, but there are still a significant number of transmasculine individuals undergoing hormone therapy who do not wish to discontinue it but may consider their fertility in the future. Therefore, we believe that, given the limited data available, this study provides valuable and necessary information.

## Conclusions

In a cohort of transmasculine people using different androgen preparations who underwent gender-affirming surgery, no differences were observed in the ovarian follicular population or the stromal tissue. Nevertheless, significant differences were observed in the hormonal status after surgery although testosterone levels were maintained in the reference range and no modification dosage was needed.

## Data Availability

The raw data supporting the conclusions of this article will be made available by the authors, without undue reservation.
